# Effects of Taylor Spatial Frame on tumors and tumor-like lesions with pathological fractures of lower extremities

**DOI:** 10.12669/pjms.342.14920

**Published:** 2018

**Authors:** Zhongbing Liu, Genling Tang, Shuguang Guo, Bin Cai, Qingsong Li

**Affiliations:** 1 Zhongbing Liu, Department of Orthopedics, Affiliated Taizhou People’s Hospital of Nantong University, Taizhou 225300, Jiangsu Province, P. R. China; 2 Genling Tang, Department of Orthopedics, Affiliated Taizhou People’s Hospital of Nantong University, Taizhou 225300, Jiangsu Province, P. R. China; 3 Shuguang Guo, Department of Orthopedics, Affiliated Taizhou People’s Hospital of Nantong University, Taizhou 225300, Jiangsu Province, P. R. China; 4 Bin Cai, Department of Orthopedics, Affiliated Taizhou People’s Hospital of Nantong University, Taizhou 225300, Jiangsu Province, P. R. China; 5 Qingsong Li, Department of Orthopedics, Affiliated Taizhou People’s Hospital of Nantong University, Taizhou 225300, Jiangsu Province, P. R. China

**Keywords:** Bone tumor, Complication, Locking plate, Taylor spatial frame, Tumor-like lesion

## Abstract

**Objective:**

We aimed to evaluate the clinical effects of Taylor spatial frame (TSF) on tumors and tumor-like lesions complicated with pathological fractures of the lower extremities.

**Methods:**

Eighty-two patients admitted from September 2013 to January 2015 were selected. Forty-two cases were included in Group-A to receive TSF fixation and forty were included in Group-B to receive locking plate fixation. The surgical time, intraoperative blood loss, postoperative healing rate of primary incision, incidence rate of complications, hospitalization stay length, and fracture healing time as well as rate of excellent and good Enneking scores one year after surgery were compared.

**Results:**

The intraoperative blood losses of Group-A and Group-B were (150.0±6.5) ml and (201.9±7.4) ml respectively (P<0.05). The surgical times were (77.3±8.9) minutes and (96.5±5.9) minutes respectively (P<0.05). The postoperative rates of complications in the two groups (4.76% vs. 10.00%) were similar (P>0.05). The primary incision healing rates of Group-A and Group-B were 97.62% and 82.50% respectively. The hospitalization stays were (15.7±0.9) days and (15.2±0.7) days respectively (P>0.05). The fracture healing times were (30.1±2.1) weeks and (32.4±2.2) weeks respectively (P<0.05). The rate of excellent and good Enneking scores one year after surgery was 97.61% in Group-A and 95.00% in Group-B (P>0.05).

**Conclusions:**

Tumors and tumor-like lesions complicated with pathological fractures of the lower extremities can be effectively treated by TSF.

## INTRODUCTION

Bone tumor is one of the common bone diseases, seriously endangering human health. Primary bone tumors occur mostly in the proximal tibia, distal femur and proximal humerus metaphysis,^1^ which are often accompanied by pathological fractures, deformities and large bone defects, causing limb dysfunction and limited mobility to seriously affect the daily life of patients. Orthopedists are challenged by not only tumors, but also reconstruction of bone defects after tumor resection, limb lengthening and deformity correction. In 1994, Taylor JC et al. first modified the Ilizarov external fixation system to create the Taylor spatial frame (TSF).^2^ Six deformity parameters (DPs), three frame parameters (FPs), and four mounting parameters (MPs) were measured. The framework was combined with a software program to input the parameters and to adjust struts according to the generated electronic prescription. It can be used to treat various fracture displacements, complex deformities, limb lengthening and bone defects.^3^-^5^

## METHODS

In this study, we selected patients with tumors and tumor-like lesions complicated with pathological fractures of the lower extremities treated in our hospital from September 2013 to January 2015. The patients were divided into two groups and treated with TSF and locking plate respectively after lesion removal and inactivation as well as allograft bone transplant, aiming to compare and to analyze the surgical time, intraoperative blood loss, hospitalization stay length, postoperative complications, primary incision healing rate and rate of excellent and good Enneking scores^6^ one year after surgery.

### Materials

**Fig.1:**
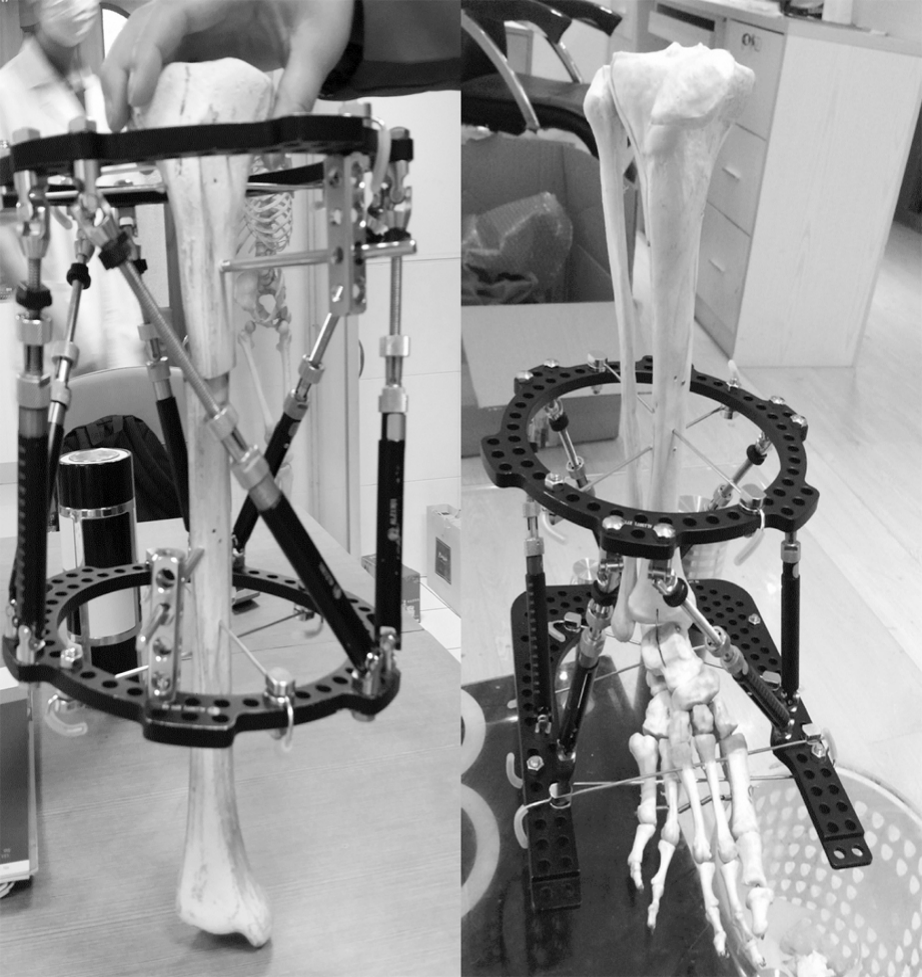
Model of TSF.

Allogeneic bone repair materials were manufactured by Beijing Daqing Biotechnology Co., Ltd. (China). TSF (Fig.1) was produced by Tianjin Xinzhong Medical Devices Co., Ltd. (China). Titanium alloy locking plate was manufactured by Shandong Weigao Orthopedic Materials Co., Ltd. (China).

### Baseline clinical data

A total of 82 patients with tumors and tumor-like lesions complicated with pathological fractures of the lower extremities treated in our hospital were randomly divided into Group-A and Group-B. This study was approved by the ethics committee of our hospital, and written informed consent was obtained from all patients.

There were 42 patients in Group-A, receiving TSF treatment after lesion removal and inactivation as well as allograft bone transplant. Group-A included 19 females and 23 males aged between 16 and 80 years old, (32.5 ± 5.9) on average. The disease courses ranged from 6 to 18 months, (10.3 ± 4.9) on average.

### Tumor sites

Seventeen cases were in the lower femur, 11 cases in the upper tibia and 14 cases in the lower tibia. All the cases were pathologically diagnosed. Pathological types of tumors: nine cases of aneurysmal bone cyst, 11 cases of simple bone cyst, eight cases of bone fibrous dysplasia, nine cases of enchondroma, three cases of non-ossifying fibroma and two cases of eosinophilic granuloma.

There were 40 cases in Group-B, receiving locking plate treatment after lesion removal and inactivation as well as allograft bone transplant. Group-B included 22 females and 18 males aged between 16 and 52 years old, (35.0 ± 7.5) on average. The disease courses ranged from 5 to 16 months, (8.6 ± 3.4) on average.

### Tumor sites

Thirteen cases were in the lower femur, 9 cases in the upper tibia and 18 cases in the lower tibia. All the cases were also pathologically diagnosed as six cases of aneurysmal bone cyst, 7 cases of simple bone cyst, 11 cases of bone fibrous dysplasia, seven cases of enchondroma, 8 cases of non-ossifying fibroma and one case of eosinophilic granuloma. The two groups had similar baseline clinical data (P>0.05).

### Inclusion Criteria

Patients with benign tumors and tumor-like lesions complicated with pathological fractures of the lower extremities; aged between 16 and 60 years old; surgery was performed by the same surgeon.

### Exclusion Criteria

Patients complicated with malnutrition, diabetes or osteoporosis; patients who were unwilling to cooperate or could no longer be contacted in follow-up.

### Preoperative preparation

Before surgery, the affected extremity was fixed by bone traction or plaster, and medical history was inquired thoroughly. A comprehensive orthopedics examination was made to determine and to detail record the characteristics of the affected extremity. Tumor characteristics were determined by pathological puncture and biopsy before surgery. Preoperative examination was improved by collecting frontal and lateral 1:1 X-ray^7^ and CT measurement data.

### Collection of Parameters

Deformity characteristics and measured DPs were not the same for different reference epiphyses. Although different reference epiphyses were chosen, the final results of deformity correction were identical.^8^ Uniform and characteristic reference epiphyses helped simplifies and understand permanent anatomical landmarks such as the patella and ankle joint. Forward displacement, lateral displacement, axial displacement, forward angulation, lateral angulation and axial angulation were collected through image measurement.^9^,^10^

### Selection of rings and struts

U-type foot ring was selected for feet and ankles, half ring was used for thighs and full ring was chosen for shanks.^11^ Fixing pins can be classified into Kirschner pin, Steinmann pin, osseous pin, screw pin and olive pin. For young adult patients and those with long treatment time and large bone defects, more pins that were thicker and had larger diameters should be selected so as not to be broken or bent after surgery.^12^

### Design of pinning planes for the lower extremities

Pinning planes were designed before surgery, and pins were inserted from an appropriate position and cross-section. The major blood vessels and nerves were circumvented, and tendons were also protected by entering through the intermuscular space. Before surgery, pinning planes were designed according to the method of Faure C et al.^13^, dividing the safe areas of the tibia, fibula and femur into 11 cross-sections and five groups.

### Surgical methods

### Group-A

After anesthesia, patient position was adjusted, drape was disinfected, a surgical film was pasted, and an appropriate surgical approach was selected to fully expose tumor lesions. A window was opened by an osteotome or electric drill, with a size that ensured complete removal of tumor under the direct view. Tumor tissues within the lesion cyst were removed with appropriate types of curettes in different directions and perspectives, and particular attention was paid to blind corners, sclerotic bones and bone ridges surrounding the cyst wall.^13^ All procedures followed the principles of tumor-free operation to avoid the local implantation and metastasis of tumors.^14^ The tumor wall was rinsed with a large amount of saline, dipped dry with gauze, inactivated by 95% ethanol for 5-10 minutes and electrocoagulation, and ground to normal cortical bone by an abrasive drilling. The medullary cavity was cleaned. Hemostasis was important during surgery. After tumor lesions were completely removed, conventional allograft bone grafting was performed in the bone defect area.^15^ A hemostatic gauze and 1-2 drainage tubes were placed, and the incisions were sutured layer by layer after the devices and accessories were carefully checked. Before installation of TSF, osseous pins with different types and diameters were chosen according to loadings and sites to provide a stable support force after surgery and to prevent postoperative stress fracture or bending.^16^ The major blood vessels and nerves 3-5 cm away from the edges of lesion were circumvented, and pinning was performed through the planes designed above. The skin was cut open (1cm) by a sharp knife at the pinning point, and the fascia and subcutaneous tissues were dissected bluntly to insert pins vertically to the bone surface. After pinning, the bleeding point was hemostatized by compression. According to preoperative design, the rings, struts and other components were assembled. Using a C-arm, the broken end of fracture was relocated by fast sliding and locking the strut. The end of fixing pin was trimmed. The mouth of pinning channel was dressed by ethanol and excipients, rings were retained, and each strut was marked.^17^

### Group-B

After anesthesia, an appropriate position was taken, the drape was disinfected, a surgical film was pasted, and an appropriate surgical approach was selected to fully expose tumor lesions. All procedures must follow the principles of tumor-free operation to avoid the local implantation and metastasis of tumors. After tumor was completely removed, the tumor wall was inactivated by electrocoagulation and 95% ethanol, rinsed with a large amount of saline and dipped dry with gauze. The broken end of fracture was stabilized with a bone forceps and relocated. Subsequently, a proper locking plate was used, on which holes were drilled successively, with their depths measured. Then the plate was fixed by screwing locking bolts. After exploration using a C-arm, hemostatic gauze and 1-2 drainage tubes were placed, and the incisions were sutured layer by layer after the devices and accessories were carefully checked.

### Postoperative treatment of TSF

After surgery, the pinning channel was kept clean and dry, and wiped with ethanol^18^, once in the morning and once in the evening. The pinning site was wrapped with gauze to prevent infection. The patients were required to elevate the affected extremities after surgery to relieve swelling. Swelling extremities were prone to compression by rings, with the risk of osteofascial compartment syndrome, so they were timely observed after surgery, and the rings should be replaced with larger ones when necessary. The pinning channels were checked regularly to check whether the pins were loose, and if so, they should be withdrawn timely before making a new channel.

### Postoperative treatment of patients

All patients were subjected to prophylactic use of antibiotics for 48 hour after surgery. The vital signs, incision dressing and volume of drainage were observed. Blood routine and biochemical examinations were conducted on the second day after surgery. X-ray films were obtained 48-72 hour after surgery, and the patients were extubated when the volume of drainage <50 ml. They were required to take isotonic exercises of muscles of the lower extremities within 24 h after surgery, and to avoid venous thrombosis induced by long-term bed rest. In addition, they were allowed to sit up 2-3 days after surgery. Group-A was allowed to walk with a crutch 3-5 days after surgery, and Group-B did so 7 days after surgery. X-ray plain films were collected 3, 6 and 12 months after surgery respectively.

### Postoperative follow-up

All patients were followed up after surgery. The follow-up time was 20.3 months on average (12-29 months) in Group-A and 7.1 months on average (12-28 months) in Group-B. Functions of the affected extremity one year after surgery were scored referring to the Enneking scale (30 points in total, maximum 5 points and minimum 0 point for each item, excellent for the total score higher than 23 points, good for the score between 15 and 22 points, and poor for the score lower than 14 point).^6^

### Statistical analysis

All data were analyzed by SPSS20.0 (IBM, NY, USA). The categorical data were expressed as mean ± standard deviation (s). The surgical time, intraoperative blood loss, hospitalization stay length and fracture healing time were compared by the *t* test. The postoperative primary incision healing rate, postoperative incidence rate of complications and rate of excellent and good Enneking scores one year after surgery were compared by the t^2^ test. P<0.05 was considered statistically significant.

## RESULTS

### X-ray films for TSF fixation

**Fig.2:**
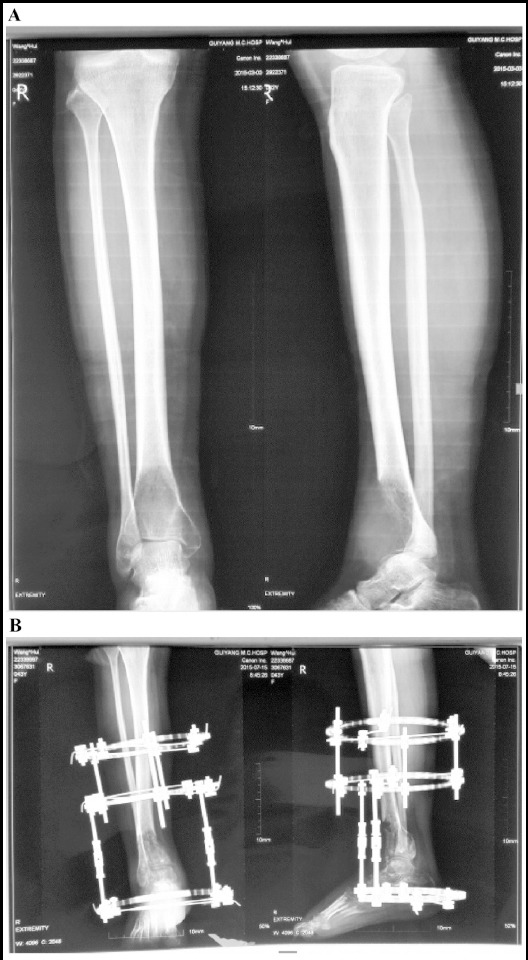
Representative X-ray films before (A) and after (B) TSF fixation.

The representative X-ray films before and after TSF fixation are shown in Fig.2.

### Intraoperative blood loss and surgical time

The intraoperative blood losses of Group-A and Group-B were (150.0±6.5) ml and (201.9±7.4) ml respectively, which were significantly different (P<0.05). The surgical times of Group-A and Group-B were (77.3±8.9) min and (96.5±5.9) minutes respectively, between which the difference was statistically significant (P<0.05).

### Postoperative complications and primary incision healing

The postoperative rates of complications in the two groups (4.76% vs. 10.00%) were similar (P>0.05). The primary incision healing rates of Group-A and Group-B were 97.62% and 82.50% respectively, which were significantly different (P<0.05).

### Hospitalization stays length and fracture healing time

The hospitalization stays of the two groups were (15.7±0.9) days and (15.2±0.7) days respectively (P>0.05). The fracture healing times of Group-A and Group-B were (30.1±2.1) weeks and (32.4±2.2) weeks respectively, between which the difference was statistically significant (P<0.05).

### Enneking scores one year after surgery

The rate of excellent and good Enneking scores one year after surgery was 97.61% in Group-A and 95.00% in Group-B, without a significant difference (P>0.05).

## DISCUSSION

Tumor lesions are often found by X-ray films in hospital to treat pathological fractures due to physical activities, participating in sports or accidental tumble.^19^,^20^ Tsuchiya H et al. reported that in 43 patients with limb bone tumors, 19% needed correction of deformity, 12% needed limb extension, 47% required correction and extension, and about 25% required bone transport.^21^ Locking plate can only be used to correct displacement from the 2-dimension perspective, which is difficult to deal with the above condition.

In 1994, Taylor et al. first modified the Ilizarov external fixation system to create TSF. The concept of deformity correction proposed by Dror made TSF application more mature.^22^ The axial strength of TSF is 1.1 times that of Ilizarov frame, 2.0 times in bending strength and 2.3 times in torque strength. Combined with a computer program, the accuracy can reach 1/1000000 inch and 1/10000°.^3^ TSF can provide continuous and solid support for bone stump and maintain it stable during limb movements, allowing the patient to walk early for functional exercises.

The intraoperative TSF installation is simple and fast, which can correct displacement by sliding struts. In this study, TSF significantly shortened the surgical time. TSF fixation requires a relatively small surgical incision, which only needs full exposure of the lesion. In contrast, locking plate needs to be placed by extending the incision. TSF crossed the bone defect area and was fixed at both ends, which was less traumatic, without needing incision extension or extensive dissection of muscles and periosteum. Moreover, the TSF group had significantly reduced intraoperative blood loss.

TSF was used in surgery to fix the fracture ends. By adjusting struts, the spatial positions of proximal and distal rings were changed to correct displacement from the three-dimensional perspective.^23^ In this study, there was one case of fracture redisplacement in the locking plate group, resulting in deformed healing. Two patients of the TSF group were unsatisfied with the reduction, who was then subjected to whole residual deformity correction program by remeasuring data to achieve functional reduction finally.

Pinning channel infection is the most common complication during external fixator treatment. Rozbruch S et al. studied 122 TSF cases and found the infection rate was 4.91%.^24^ Group-A in this study had one case of exudation from pinning channel, with an incidence of 2.38%. The patient recovered after strengthening the dressing. Besides, one case suffered from vascular injury. The patient was then treated through reducing swelling and improving circulation until hematoma was gradually absorbed.

## CONCLUSION

Tumors and tumor-like lesions complicated with pathological fractures of the lower extremities can be effectively treated by TSF. Compared with locking plate, TSF can significantly decrease intraoperative blood loss and surgical time, as well as significantly promote primary incision healing and fracture healing. However, whether there are other complications and disadvantages needs to be further studied in clinical practice.

### Authors’ Contributions

**ZL** designed this study and made substantial revisions to the manuscript.

**GT, SG, BC & QL** collected and analyzed clinical data.
